# Precuneus brain response changes differently during human–robot and human–human dyadic social interaction

**DOI:** 10.1038/s41598-022-14207-9

**Published:** 2022-08-30

**Authors:** Nicolas Spatola, Thierry Chaminade

**Affiliations:** 1 Artimon Perspectives, Paris, France; 2 Institut de Neurosciences de La Timone, UMR 7289, Aix-Marseille Université—CNRS, Marseille, France

**Keywords:** Psychology, Human behaviour

## Abstract

Human–human interactions (HHI) and human–robot interactions (HRI) are compared to identify differences between cognitive processes reflecting bonding in social interactions with natural and artificial agents. We capitalize on a unique corpus of neuroimaging data (fMRI) recorded while participants freely discussed with another human or a conversational robotic head, in order to study a crucial parameter of human social cognition, namely that social interactions are adaptive bidirectional processes that evolve over time. We used linear statistics to identify regions of the brain where activity changes differently when participants carry out twelve one-minute conversations, alternating between a human and a robotic interlocutor. Results show that activity in the posterior cingulate cortex, a key region associated with social cognition, increases over time in HHI but not in HRI. These results are interpreted as reflecting a process of strengthening social bonding during repeated exchanges when the interacting agent is a human, but not a robot.

## Introduction

We, Humans, are intrinsically social beings. Throughout our evolution, social interactions have structured our behaviors, our brains and ultimately our environment^1^. Thanks to technology, our species continuously diversifies the way people engage with fellow humans, and more recently, with artificial agents such as social robots. In addition to providing new forms of social interaction, these artificial agents also offer opportunities to investigate human social cognition^2–4^. In the past decade, the emergence of paradigms comparing Human-Robots Interactions (HRI) to Human–Human Interactions (HHI)^2–6^ opened new avenues for studying social interactions and to better understand how the human social brain, initially devoted to HHI, adapts to HRI. However, crucial questions remain about how we engage in HRI, and in particular how our tendency to attribute mental properties to other agents apply to robots. In the present study, capitalizing on a unique fMRI corpus, we investigate how the human brain activity is differently affected by repeated interaction with a human and a robot.

To explain human social cognition, the “cognitive systems theory” posits the existence of a “social cognition system” and a “physical cognition system”^7–10^. Numerous neuroimaging studies provide evidence for extended different neural networks specialized in each of these systems^9,11^. This distinction was theorized decades ago by philosopher Daniel Dennett, asserting that we adopt an intentional stance (attributing mental states, such as intention, to make sense of an agent's actions, e.g. she takes her car and drive because she wants to go to the restaurant) only when interacting with fellow humans, and a design stance (explaining the agent's actions through functional causation, *e.g.* a car is propelled by the chemical energy contained in gasoline) when interacting with mechanical agents because of a lower level of abstraction in explaining an observed action. Results from social cognitive neurosciences latter confirmed that the activation^12^ of the social brain was significantly increased when interacting with humans compared to robot or computer agents^13^, yet it is possible to adopt, to a certain extent, an intentional stance toward social robots^14,15^.

However, these investigations usually consider the interaction as a stable process from the beginning to the end of an experiment. In other words, the stance we would adopt at the onset of an interaction is thought to remain stable until its end. This assumption contradicts the way humans naturally engage in interactions with others. During an interaction, individuals’ perception of the interactor evolves according to the characteristics of the interaction, in particular the representation of the other agent one builds online^16,17^. Therefore, the representation of the interactive agent evolves over time instead of remaining exclusively restricted to either the physical or the social cognition system (respectively, a design and an intentional stance). This is likely to be untrue. Here, we posit that processes involved in HHI and HRI could vary differently depending on the past history of the interaction at the time of measurement. For instance, one could first adopt automatically a default stance, when the human and robot agents are unfamiliar, that would be common to both agents: the intentional stance. As the interaction unfolds, the stance would adapt according to the nature of agent, a human or a robot, the individual is interacting with, through an iterative process. Importantly, this implies that both interlocutors behave differently, meaning that their behavior displays information about their nature, as it the case with the current data. The influence of time should be lessened when the nature of the agent is induced by contextual information rather than perceivable cues. This hypothesis, of different changes in how the adopted stance adapts during repeated human–human interactions (HHI) and human–robot interactions (HRI) is tested here by analyzing a corpus of human–human and human–robot natural conversations that includes fMRI brain data as physiological recordings reflecting cognitive processes^13^.

This corpus allows us to address the effect of repeating similar human–human and human–robot interactions. Importantly, these interactions were unconstrained and the behaviors were unique for each participant and each trial, hence also between the two agents. We analyzed whether the local brain physiological correlates of these interactions evolved differently according to the nature of the interlocutor. We were able to identify differences in how physiological activity recorded with fMRI when engaging in a natural social interaction with an natural or an anthropomorphic artificial agent evolves in brain regions that are not directly related to the behavior itself, an unconstrained bidirectional conversation^18^.

**Figure 1:**
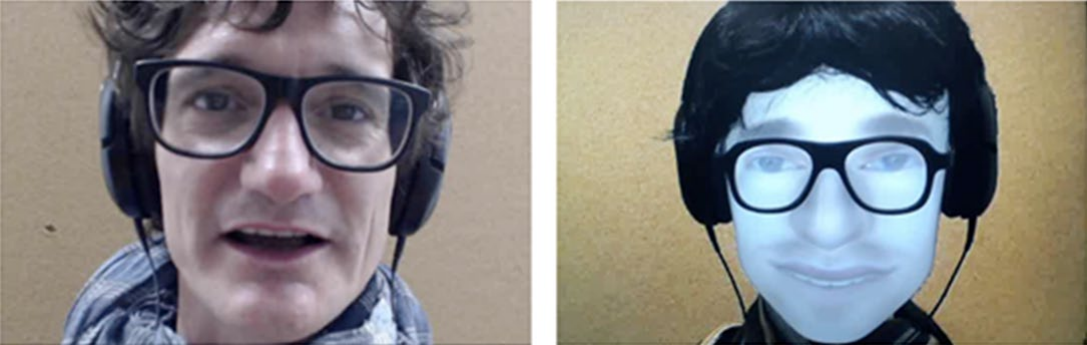
Single frames extracted from the live video feeds projected on a screen visible on a mirror positioned in front of the scanned participants’ eyes, with the Human (left) vs Robot (right) male conversant. Informed consent from participant for publication was obtained.

In the experiment, participant comfortably lying in an fMRI scanner discussed with a human conspecific and a robotic device resembling a human head and face (Fig. 1). They believed the robot to be autonomous, while it was in fact controlled at distance by the human agent through a simple remote interface with a limited number of utterances to select from by pressing virtual buttons on a touchscreen. They had to discuss images representing “super-heroes” and “rotten fruits” supposedly designed for an advertisement campaign, during twelve trials (≈ 1 min/trial) for each interlocutor. Overall, from beginning to end, the acquisition of the 24 trials used in the analyses, 12 for each agent recorded alternatively, lasted around 40 min for each participant. This paradigm of online natural discussion comparing human and robot agents is the first of its kind in the sense that control of the behavior is limited to the instruction to “freely discuss the images”, while recordings of the behavior are used to extract variables for later analysis (see for example^19^ for an analysis of different linguistic alignments between HHI and HRI and their neurophysiological correlates).

## Results

### Analytical approach

A linear statistical parametric model (SPM), defining each one-minute interaction trial independently, was computed for each participant separately using SPM12^20^, allowing to estimate whole-brain maps of ß-weights corresponding to linear changes of activity for each individual trial. An average of estimated ß-weights was extracted, using MarsBar toolbox^21^, for each trial and participant, in regions of interest covering the whole brain. Two-hundred forty-six regions were taken from a connectivity-based parcellation of the brain^22^, complemented by an additional mask of the hypothalamus that was developed for a previous study of social interactions^13^ as it was not available in the original parcellation. Two vectors of 12 mean ß estimates were obtained for each participant and each region, corresponding to the 12 HHI and HRI trials. We conducted a multivariate linear mixed model analysis of these ß estimates defined by the type of agent (human vs. robot), the 12 consecutive trials of interaction (that represent time) and the 247 Regions Of Interest (ROIs). The use of a single model including all ROIs was used to control for repeated measures across brain regions, Participants were introduced in the model as a random effect (see “Methods” section). The results presented here focus on significant interactions between the nature of the agent and the trials in all regions of interest, at the conservative threshold of *p* < 0.001.

### Analyses

**Figure 2:**
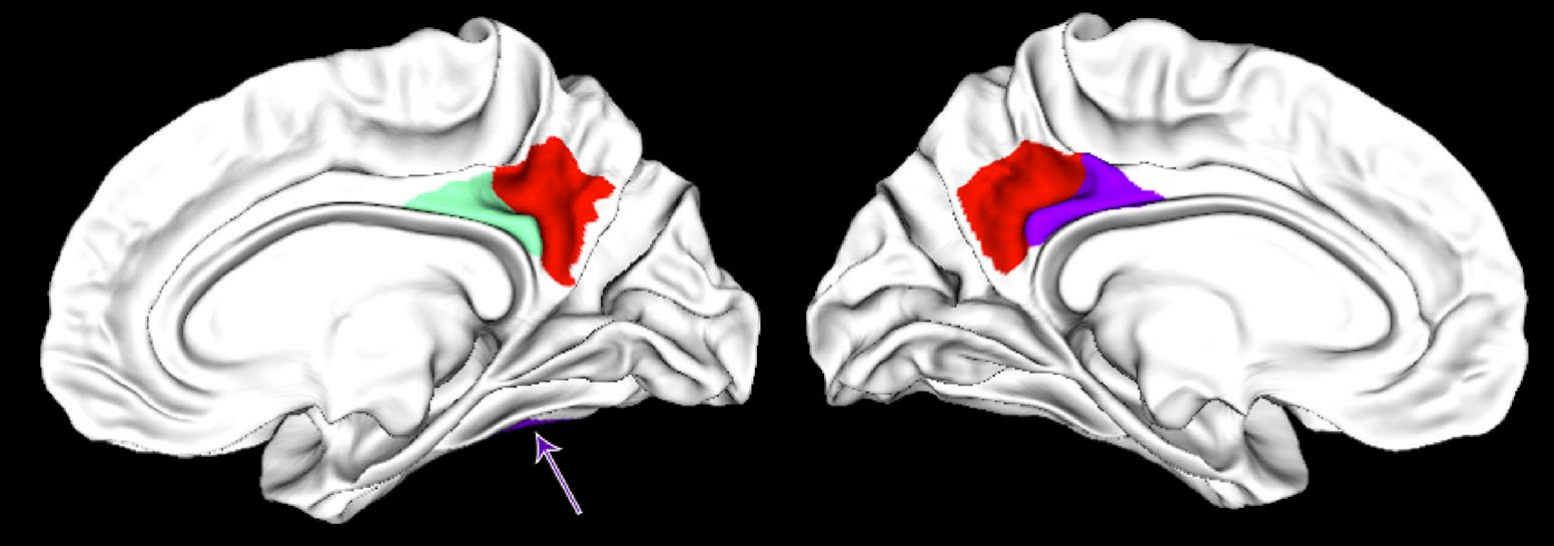
Regions of interest in the posterior cingulate cortex (dorsal bank of the sulcus in red, ventral bank in green and purple for the right and left hemispheres respectively) where the bold signal is differently affected by time depending on the nature of the agent. The right ventral fusiform region can be seen in purple on the bottom of the left brain (pointed by an arrow).

**Figure 3:**
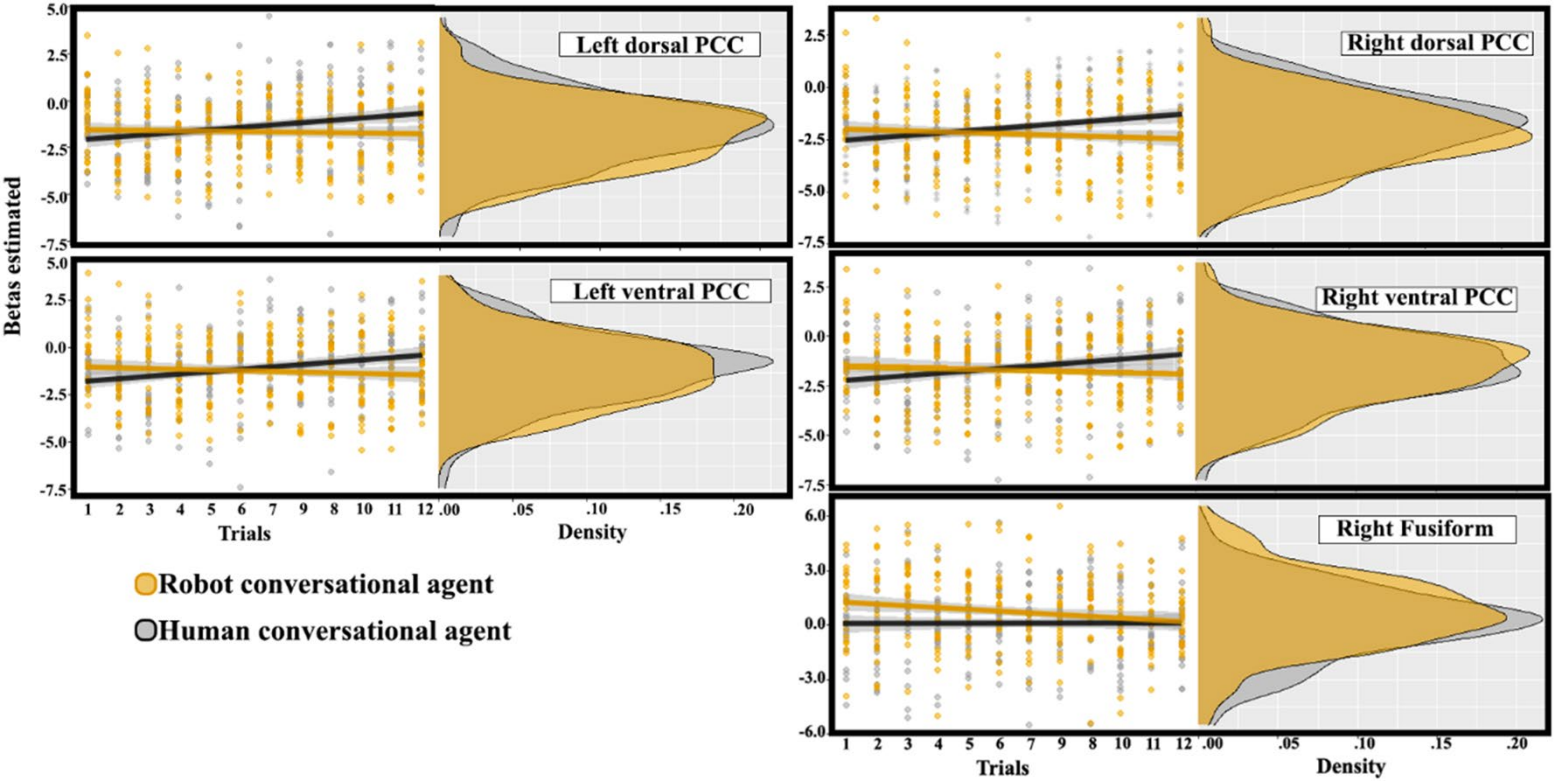
BOLD signal as a function of the type of interactor (human vs robot) and consecutive trials (corresponding to time) in the dorsal and ventral Posterior Cingulate Cortex (PCC) bilaterally.

Interestingly, in the main analysis including both human and robot conditions as independent variables, we only found five cortical regions in which the ß estimates significantly evolved differently through time for the human and the robot agent. Four of these formed two clusters around the sulcus in the Posterior Cingulate Cortex (PCC) bilaterally, as illustrated in Fig. 2. All four regions yielded similar statistical results (dorsal PCC, upper bank of the cingulate sulcus and medial wall above: left: *B* = − 0.14, *t*(20) =  − 3.30, CI_95%_ [− 0.23; − 0.06]; right: *B* = − 0.15, *t*(20) =  − 3.48, CI_95%_ [− 0.23; − 0.06]; ventral PCC, ventral bank of the cingulate sulcus and medial cingulate gyrus: left: *B* = − 0.16, *t*(20) =  − 3.82, CI_95%_ [− 0.24; − 0.08]; right: *B* = − 0.15, *t*(20) =  − 3.67, CI_95%_ [− 0.24; − 0.07]; all *p*s < 0.001). When considering HHI and HRI separately, an increase of the ß estimates over time was only found for the HHI condition in these four areas (Fig. 3; dorsal PCC: left: *B* = 0.11, *t*(20) = 4.01, CI_95%_ [0.06; 0.18]; right: *B* = 0.10, *t*(20) = 3.45, CI_95%_ [0.04; 0.16]; ventral PCC: left: *B* = 0.12, *t*(20) = 4.09, CI_95%_ [0.06; 0.18]; right: *B* = 0.11, *t*(20) = 3.83, CI_95%_ [0.05; 0.17]; all *p*s < 0.001), with no significant effect of trial number (time) in HRI (dorsal PCC: left: *B* = − 0.02, *t*(20) =  − 0.52, CI_95%_ [− 0.07; 0.04]; right: *B* = − 0.04, *t*(20) =  − 1.36, CI_95%_ [− 0.10; 0.02]; ventral PCC: left: *B* = − 0.04, *t*(20) =  − 1.30, CI_95%_ [− 0.10; 0.02]; right: *B* = − 0.04, *t*(20) =  − 1.32, CI_95%_ [− 0.09; 0.02]; all *p*s > 0.001). The brain activity increases with time in these posterior medial regions of interest bilaterally when interacting with the human. Interestingly, none of these regions were significantly affected by the nature of the interacting agent without taking into account the time variable. In other words, without considering the effect of time on the BOLD signal, one would have concluded that activity in this core area of the social brain didn't differ between HHI and HRI, as illustrated by the density plots shown on the left side of each panels in Fig. 3. We also performed *t*-tests between HHI and HRI for each ROI and trial number, and only a few yielded significant results. This confirms the approach chosen for the statistical analysis: behaviors vary trial per trial, it is therefore not expected that the effect of the agent would be significant at the same time points across participants; yet the linear approach spanning the whole duration of the experiment and using trial number as a proxy for time confirms that it is the repetition of trials that affected brain activity in the precuneus, instead of one (or a few subset of) specific trial(s) common to all participants.

A significant interaction between the agent and the trial number was found in a region of interest corresponding to the right fusiform gyrus (*B* = − 0.14, *t*(20) =  − 3.64, CI_95%_ [− 0.22; − 0.07], *p* < 0.001). The pattern was different from regions of the previous cluster, as the activity didn’t significantly changed in HHI (*B* = − 0.02, *t*(20) = 0.73, CI_95%_ [− 0.03; 0.07], *p* = 0.466), but decreased with time in HRI (*B* = − 0.12, *t*(20) =  − 4.25, CI_95%_ [− 0.17; − 0.06], *p* < 0.001).

## Discussion

According to the analysis presented here, time, operationalized as trial number in a corpus comprising consecutive trials of natural social interactions, affects the activity differently depending of the nature of the interlocutor, a human or a robot, in five brain regions. The right fusiform cortex had the following dynamic: activity significantly decreased with time for the robot, while changes were not significant for the human. Given that that the fusiform gyrus activity is associated with the visual processing of faces, and that the response to the robot, but not to the human, significantly reduced with time, our interpretation is that participants became familiar to the novelty of the previously unknown robotic face^23,24^.

More directly related to our hypotheses, four other regions formed a cortical cluster in the posterior cingulate cortex (PCC) bilaterally. The left and right regions on the ventral bank of the posterior cingulate sulcus correspond to Brodmann area 31, and the dorsal ones, to Brodmann area 23^22^. Reference to Brodmann areas can be relevant because, as they are defined based on their cytoarchitectonic properties^25^ they correspond to homogeneous areas of neuronal processing. The brain parcellation used here defines homogenous areas on the basis of their functional and anatomical connectivity, which is believed to detail further the cytoarchitectonics used originally by Brodmann^26^. Across primates species including humans^27^, areas 23 and 31 of the PCC are characterized by an increase of size and of proportion of superficial layers interpreted as "*a progressive structural differentiation permitting an increasing amount of intracortical information-processing capacities"*^25^. Importantly for the current analysis, they have not been associated with speech in any specific way, so that it is unlikely that their finding in the current analysis could be explained by the differences in verbal behaviors of the two interlocutors. An additional argument discarding such an interpretation is that they were not identified in direct contrasts between the two agents (described in Figs. 2 and 3 and in Rauchbauer et al.^13^), a result conformed by the density of estimates on the left of each PCC plot in Fig. 2. Altogether, these arguments favor the interpretation that the present finding reflects a mechanism associated with dynamical changes associated with repeated social interactions instead of lower sensory differences between the human and robot agents.

Involvement of the PCC has been reported in a number of tasks pertaining to social cognition^27^, such as self-reflection^27^, the sense of agency (attributing executed or imagined actions to oneself^28^ or to another agent^29^), familiarity, of people^30^ or places and objects^31^), or emotional judgements about words^32^ or faces^33^. The PCC is systematically activated in tasks that require self-generated thoughts, including self-referential processing, episodic or autobiographical memory, future thinking, mentalizing, spatial navigation, and conceptual processing^34–36^. One common theme to all these tasks, suggested by the connectivity of the region^37^ is to bring together autobiographical memory, as it is directly connected to parahippocampal areas involved in episodic memories^38^, and emotional processing from adjacent cingular areas. It is also close and connected to early visual areas, in particular from the ventral stream, a possible explanation of its involvement in mental imagery^29,37^. The PCC is also the central node of the Default Mode Network (DMN), originally defined by an increased connectivity during rest compared to task-oriented activity^39,40^. It is now well established that the DMN and the “social brain” areas overlap largely^41^, one hypothesis being that DMN activity takes place when the brain is free to monitor both the environment and inner world without task constraints^27^. Altogether, it was proposed that the PCC plays a central role in adapting behaviors to changes in the world: it acts as a gateway controlling whether and which task-positive network should be engaged as a function of external information on the one hand, and self-episodic memory and emotional processing^42^ on the other hand.

The current results further the understanding of the involvement of this region by showing its activity is evolving during natural social interactions. These changes argue for a dynamic process in HHI with an increase of social engagement with the human agent as an iterative process. In contrast, in HRI, this process isn’t significantly influenced by time. While it is not possible to ascertain the origin of these different dynamics, the fact that ß estimates increase with repeated interactions are significant in the PCC areas for the human only argues in favor of an accumulation of social information with time, while the robotic device used in this experiment only provides limited social signals. These different dynamics in the PCC could represent the building of familiarity or trust and a deepening of the (emotional) engagement in the interaction restricted to the human interlocutor despite the relative short period of time involved (12 min with each agent). This result echoes the top-down control proposed by Daniel Dennett’s intentional stance that postulates that we would rather adopt a design stance when facing a machine^12^. As such we expect a drop of PCC activity on the long-term in HRI as theorized in the initialization, familiarization and stabilization framework^43^. In this framework, during the initialization stage the novelty effect^44^, the lack of knowledge required to develop a specific representation of the robot^45^, the will to efficiently interact with this new agent)^46^ and as a consequence, the tendency to reproduce the way we engage with humans on HRI^47^ would lead to the reliance on a common default stance. This initialization stage would be followed by a drop in mentalistic attributions when the initial apparent complexity of the robot is contradicted by its limited social abilities: the default, initial, intentional stance is kept for the human, but replaced by the mechanical stance for the robot.

The cluster of four PCC areas showed a significant increase of linear estimate associated with the time spent interacting with the human; the fact that this region is a major controller of the social brain network^48^ suggests that social cognitive representations are updated during repeated interactions with a fellow human. In contrast, no linear changes with time, increase or decrease, is found when the interacting partner is the robot. This contrast between brain dynamics during HHI and HRI interactions in the PCC, suggesting that the robot is not considered a valid social partner, constitutes an important starting point for further investigations, despite a number of limitations. The most obvious limitation concerns the poor social cues depicted by the conversational robot used in this experiment. The limited social cues provided by the facial expressions and the speech, intonations, is clearly a limitation of the experiment. While intended initially to increase experimentally the contrast between interactions with the human and the robot agent, we acknowledge it obviously limits the generalizability of the present results to human–robot interactions in general. It should be noted though that this is true of all studies of human–robot interactions, but is unfortunately rarely mentioned as a fundamental factor to interpret the results. Meanwhile, we would like to highlight the larger perspectives of this research program. One of them is to find objective neurophysiological markers of the social acceptability of an artificial agent during repeated interactions. We focused here on the effect of repeated exposure and found that the dynamics of posterior areas of the cingulate cortex offer a promising metric of such social history. In parallel, analyses focused on identifying neurophysiological markers of verbal alignment with, empathic response towards, or attribution of intentions to, humanoid robots, need to be performed to identify brain markers associated with other dimensions of social bonding.

A final perspective is to identify the respective role of perceptual and contextual cues on the acceptance of artificial agents such as humanoid robots as social partners. Some authors posit that reaching a certain threshold of social complexity—for instance adding some non-monotonic (evolving through time) social behavior such as self-disclosure^49^, non-verbal behaviors such as eye contact, joint attention and emotions^50^ or social support^51,52^—robots could maintain users’ social engagement^52^. At first glance the present results argue for a negative answer, but they should be considered as very preliminary. Interacting with a human naturally entails strong bidirectional and iterative social cognition processes—“strong” is taken in relation to human–robot interactions; of course, there are multiple examples in which two individuals won’t cope well and the match will not be that “strong”; what is important is the comparison to interactions with humanoid robots. A laugh from the participant can trigger a reacting laugh in a human partner, and a joke is likely to be carried over time. But in the present experiment, such behaviors were impossible during human–robot interactions. One should say almost impossible as even though the human actually controlling the robot could refer to a previous interaction, the limited number of possible utterances available to the robot made spontaneous adapted responses quasi impossible and, in any ways, far from reproducing natural social interactions. While technical limitations are a part of the explanation, another reason was experimental. The final objective of this research program is to compare these results with those obtained with a socially optimized robot, in order to compare the effect of the contextual knowledge(or belief) about the autonomous nature of the artificial agent on the brain correlates of the interaction while controlling the quality of social cues provided by the robot. Reproducing natural interactions with an artificial device is a challenging task that is still ongoing.

In conclusion, the main objective of this research program, that pertains both to understanding the social cognitive neuroscientific bases of human social cognition and to anticipating the future of human–robot interactions, is to identify parameters of a social interaction that are influenced, quantitatively or qualitatively, by distinct aspects of the interacting agent’s individual characteristics, with a particular focus to distinguish between contextual knowledge about the nature of the robot and the social cues the robot conveys through its behavior. As mentioned earlier, this issue will be addressed directly by reproducing the same experiment but with a humanoid robot with significantly improved social competences. This should allow us to answer the key question: Can an artificial agent be considered as a human-like social partner if it acts as a human, or will it forever be considered as an artifact given our knowledge of its true nature, towards which it is impossible to adopt an intentional stance or express sincere empathy^53^?

## Materials and methods

The present research has been performed in accordance with the Declaration of Helsinki, and informed consent was obtained from each participant.

### Procedure

Twenty-five participants came to participate to the experiment, and 24 included in the analyses after one participant was excluded for insufficient task compliance (17 women, $\overline{M }$_age_ = 26.76 years, SD. = 7.96).

In order for participants to be unaware of the real objectives of the experiment, a cover story was presented to justify the experimental task. Participants believed they participated to a neuromarketing experiment investigating whether they could find the purpose of two upcoming marketing campaigns after observing three images for each campaign and discussing about them with the two partners. The experimenter introduced them to their two interlocutors for the experiment, a fellow human also participating to the experiment (in reality, an experimenter [author TC for men, a master student for women] of the same gender than the participant) and a back-projected conversational robotic head (Furhat from KTH^54^). The gender, voice and various accessories of the artificial agent were adapted to increase the resemblance between the human and the artificial agent. After participants were installed in the scanner, they underwent four sessions of approximately eight minutes of functional magnetic resonance imaging (fMRI) acquisition during which the BOLD signal (Blood Oxygen-Level Dependent) was recorded continuously, each consisting of 6 experimental trials that proceeded as follows: a picture appeared for 8.3 s, then after a 3 s pause with a white fixation cross on a black background, a live discussion of approximately 60 s took place, alternatively with the human and the robot. There were additional periods of rest, approximately 5 s, at the beginning and end of each session as well as between individual trials. Both the participant and interlocutor could hear each other in real-time, and the participant additionally saw a live video feed of the interlocutor, human or robot. In total, each participant took part in twelve one-minute conversations with the human and twelve one-minute conversations with the artificial agent. Anatomical images as well as images of the inhomogeneities of the magnetic field used in the analysis were recorded while participants were in the scanner (details in Rauchbauer et al.^13^).

### Agent speech

In each trial, the participant was instructed to initiate the discussion. The human speech was unconstrained. The robot’s production was controlled by the same confederate who was acting as the human interlocutor: it has a set of pre-recorded answers that were selected online by pressing virtual buttons on a touch-sensitive tablet. Examples of answers, some generic (e.g. “yes”, “no”, “maybe”, “I don’t know”, “Can you repeat the question?”) and others specific to an image (“It's a yellow pear”) or to the campaign (“Maybe it's a campaign to promote local fruit”) are given in Table 1. The robot text-to-speech synthesizer (Amazon Polly, voices Lea and Matthieu for French) then produced the selected utterance, with the robot’s own lip-syncing controller. Individual tuning of the recorded audio gain was performed to ensure that the volume and understandability of the confederates’ voices would be similar across participants.

**Table 1:** Example of pre-recorded sentences pronounced by the robot, grouped according to their function in the conversation: presentations, generic answers, descriptions of an image (a pear in the series “rotten fruits”), and exchanges on the message of the advertising campaign.

French	English
Bonjour	Hello
Salut	Hi
Je m’appelle Furhat	My name is Furhat
Comment ça va ?	How's it going?
Bien	Good
Merci	Thank you
Oui	Yes
Oui c’est possible	Yes, it's possible
Non	No
Peut-être	Maybe
C’est une poire	It's a pear
C’est une poire jaune	It's a yellow pear
La poire a l’air malade	The pear looks sick
Elle paraît faible	She seems weak
Elle semble fatigue	She looks tired
La poire semble triste	The pear looks sad
Elle n’a pas l’air contente	She doesn't look happy
Elle semble malheureuse	She seems unhappy
Peut-être que quelqu’un l’a frappé	Maybe somebody hit him
Peut-être qu’elle est malade et elle est devenue pourrie	Maybe she's sick and she's gone rotten
Tu as une idée du message ?	Do you have any idea what the message is?
C’est peut-être une campagne pour favoriser les fruits locaux	Maybe it's a campaign to promote local fruit
C’est peut-être pour manger des fruits et légumes avant qu’ils pourrissent	Maybe it's to eat fruits and vegetables before they rot
Ça pourrait être une pub pour des producteurs de fruits	It could be an ad for fruit growers

Given the lack of constraints for the human speech compared to the limited number of different utterances of the robot, there were differences in their respective productions (e.g. increased response delay; limited response repertoire; limited vocalization). It is important to stress here that in this experiment, these differences are part of the experimental question, that is incoming verbal information is representative of the nature of the agent being interacted with.

### Data preparation

The processing of fMRI data followed standard procedures. The volumes acquired represent the blood oxygenation level-dependent signal (BOLD) in 2.5 cm^3^ voxels of the brain (repetition time 1.205 s). Preprocessing entails a correction of temporal synchronization of the acquired slices, a realignment of the volumes on the first one, and a correction of the deformation due to the local distortions of the magnetic field. The Artifact Detection Toolbox (https://www.nitrc.org/projects/artifact_detect) was used to ensure that speaking by participants during the scanning didn’t yield movement artifacts that would have required exclusion of large chunk of data, which wasn’t the case with the standard threshold of 2 mm used. Note though that this is a clear limitation of the experiment: speech can’t be fully considered as natural when participants are lying supine in an fMRI scanner and required to refrain from moving their upper head (vocalizing mainly relies on the lower jaw, tongue and lips movements, themselves not known to lead to intractable artifacts in an fMRI experiment). Normalization allowed us to put the brains of all participants in the standard MNI space using the DARTEL procedure^55^. Several nuisance covariates were computed to eliminate motion artifacts, potential blood pulse and respiration artifacts, which were highly relevant in a paradigm involving speech, as well as global grey matter signal, white matter activity, and cerebrospinal fluid activity to control global signal fluctuations unrelated to the task (TAPAS toolkit^56^).

The analysis of fMRI data was first based on the general linear model implemented in SPM^20^. Each trial was modelled as a single regressor, and the images presented before each discussion were modelled as a single regressor. These regressors were convolved this the HRF. Each session was also modelled removing the main effect of whole brain BOLD signal per session in effect normalizing the signals used for the ß estimates for individuals regressors. We used a brain parcellation formed from functional and connectivity brain data, the Brainnetome atlas^22^, so that regions of interest represented sets of voxels that are homogeneous in terms of function. In each of the 246 regions of the atlas as well as in a hypothalamus mask developed in canonical MNI space^57^ (The hypothalamus is absent from the Brainnetome parcellation) the ß estimates were extracted using the MarsBAR toolbox^21^ and the set of values for the 24 participants, 12 sessions per agent and 247 regions were used for statistical analyses.

### Ethics

The project received ethical approval from the Comité de Protection des Personnes (CPP) Sud-Marseille 1 (approval number 2016-A01008-43). Written consent was obtained from all participants.

### Informed consent

Informed consent was obtained from all subjects.

## Data Availability

Transcribed linguistic data can be found on Ortolang (https://www.ortolang.fr): https://hdl.handle.net/11403/convers; fMRI group data on Neurovault (https://neurovault.org/): /collections/ASGXRWEM/; fMRI raw data can be found on OpenNeuro (https://openneuro.org/): https://openneuro.org/datasets/ds001740.
